# The Thermoluminescence Response of Ge-Doped Flat Fibers to Gamma Radiation

**DOI:** 10.3390/s150820557

**Published:** 2015-08-20

**Authors:** Siti Nurasiah Binti Mat Nawi, Nor Fadira Binti Wahib, Nurul Najua Binti Zulkepely, Yusoff Bin Mohd Amin, Ung Ngie Min, David Andrew Bradley, Roslan Bin Md Nor, Mohd Jamil Maah

**Affiliations:** 1 Radiation Research Laboratory, Department of Physics, Faculty of Sciences, University of Malaya, Kuala Lumpur 50603, Malaysia; E-Mails: fadira@siswa.um.edu.my (N.F.B.W.); najuazulkepely@um.edu.my (N.N.B.Z.); yusoffmohdamin@um.edu.my (Y.B.M.A.); rmdnor@um.edu.my (R.B.M.N.); 2 Department of Clinical Oncology, Faculty of Medicine, University of Malaya, Kuala Lumpur 50603, Malaysia; E-Mail: nm_ung@um.edu.my; 3 Centre for Nuclear and Radiation Physics, Department of Physics, University of Surrey, Guildford GU2 7XH, UK; E-Mail: d.a.bradley@surrey.ac.uk; 4 Department of Chemistry, Faculty of Science, University of Malaya, Kuala Lumpur 50603, Malaysia; E-Mail: mjamil@um.edu.my

**Keywords:** thermoluminescence, flat fibers, glow curve, reproducibility, dose response

## Abstract

Study has been undertaken of the thermoluminescence (TL) yield of various tailor-made flat cross-section 6 mol% Ge-doped silica fibers, differing only in respect of external dimensions. Key TL dosimetric characteristics have been investigated, including glow curves, dose response, sensitivity, fading and reproducibility. Using a ^60^Co source, the samples were irradiated to doses within the range 1 to 10 Gy. Prior to irradiation, the flat fibers were sectioned into 6 mm lengths, weighed, and annealed at 400 °C for 1 h. TL readout was by means of a Harshaw Model 3500 TLD reader, with TLD-100 chips (LiF:Mg, Ti) used as a reference dosimeter to allow the relative response of the fibers to be evaluated. The fibers have been found to provide highly linear dose response and excellent reproducibility over the range of doses investigated, demonstrating high potential as TL-mode detectors in radiation medicine applications. Mass for mass, the results show the greatest TL yield to be provided by fibers of the smallest cross-section, analysis indicating this to be due to minimal light loss in transport of the TL through the bulk of the silica medium.

## 1. Introduction

Thermoluminescence (TL), more definitively Thermally Stimulated Luminescence (TSL), is the phenomenon in which a selected TL medium previously exposed to penetrating radiation emits light under application of heat. Over a period of many decades, the technique has been harnessed as an effective means of evaluating the energy deposited in a medium following irradiation. The method is versatile, not least in regard to its response to a wide range of sources, including charged particles (e.g., β, proton and α-particles) as well as uncharged radiations (e.g., UV, *x*- and γ-rays, and neutrons). TSL has thus become the basis of thermoluminescence dosimetry (TLD) [1], with various application areas covered by this passive form of detection, including dating of material of archaeological interest as well as environmental exposure analysis through to evaluation of doses resulting from medical irradiations and radiation processing.

With respect to suitability, several key properties of TL media need to be established, including traps concentration, stability of the traps, efficiency of light emission arising from recombination processes, photon-energy and dose-rate dependence, reproducibility and linearity of TL yield as a function of dose. In previous work, favourable dosimetric performance has been established for doped optical fibers, sufficient to cover a wide spectrum of applications [2,3,4,5].

For silica fibers, doped up to an optimal level beyond which self-absorption occurs, the addition of a well-considered dopant to the intrinsic silica increases the number of traps, also enhancing the radiation response of the medium. In optical fibers fabricated for telecommunication purposes, dopants are routinely incorporated in the silica glass preform, such that under highly controlled circumstances the refractive index of the core of the resultant fiber will be modified to obtain low-loss light transportation [3]. In present studies, pure silica (SiO_2_) glass fibers doped with Ge have been used, the latter representing a dopant possessing the same number of outer electrons as silicon (Si), structural incorporation leading to minimal disruption but with large difference in the total number of electrons. This doped medium has been shown to exhibit TL yield significantly greater than that of telecommunication fibers doped with Al, Nd, Yb, Er, and Sm [6,7,8]. As such, there is the expectation that it can provide a sensitive system for radiotherapy dose audit, approaching or perhaps exceeding that of TLD-100, the latter being a highly popular phosphor-based system of LiF doped with Mg and Ti.

In regard to Ge-doped silica (SiO_2_) fibers for optical fibers telecommunication, by increasing the total number of electrons in the core glass, the speed of light in the core is decreased, leading to commensurate increase in refractive index. Thus, Ge is commonly used as a dopant to achieve excellent light transport performance. The addition of Ge also significantly increases the number of defect centres within the glass, fortuitously increasing the TL response in such media [4,5,9,10]. Previous dosimetric studies in this area of endeavour have made use of commercially available circular cross-section telecommunication fibers, not least because of their wide availability but also because of their advantageous response in radiotherapy dosimetry applications compared to other competing forms of passive dosimeter, TLD or otherwise [1,2,3,4,5].

**Figure 1:**
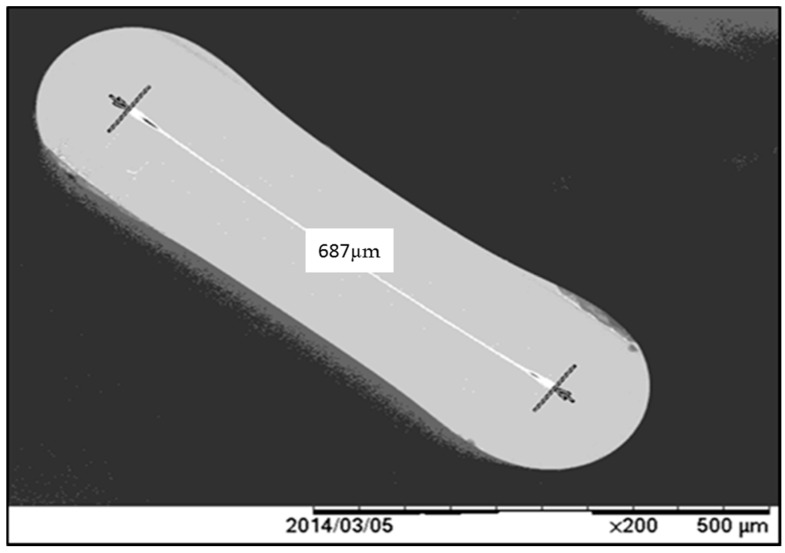
Typical cross-sectional image of a section of a flat Ge-doped optical fiber. At the centre of the fiber, indicated by the white line, is the location of the core containing the Ge dopant.

In the research reported upon herein, in an effort to increase the TL yield of the fibers use has been made of 6 mol% flat cross-section Ge-doped silica fibers (for commercially available circular cross-section Ge-doped telecommunication fibers it has been our experience that the dopant concentration is typically closer to 4 mol%). The increased dopant concentration and the flattened cross-sections of the fibers are unique features of the current study, benefitting from the facilities of the consortium collaboration of which the present group are partners, allowing fibers of various dopant concentrations, shape, and form to be fabricated. The advantage of using flattened fibers for application in medical radiotherapy has also been reported by others [4,9,10,11]; in particular, it is contended that the greater sensitivity of these radiation dosimeters in comparison with that of circular cross-section fibers stems in good part from the increased thermal contact the flat fibers enjoy with the heating plate (the so-called planchette). The intention of the study herein is to seek a flat fiber arrangement providing competitive radiation sensitivity with that of the widely used LiF:MgTi dosimeter, a dosimeter which is available in chip, disc, and rod form. Here it is important to note that the process of collapsing the initially hollow circular cross-section doped silica preform into a solid flat fiber is brought about by application of a vacuum (Figure 1, providing an example of the flat fiber shape). This has been demonstrated elsewhere to generate strained inner-contact surface defects [9,10,12]. These newly generated defects add substantially to the relative sensitivity of the fibers. With these features in mind, key TL characteristics have been investigated in this new form of fiber, including the glow curves associated with four different cross-sections of flat fiber, dose response, and reproducibility. The results have been compared against that for the well-established and highly popular phosphor-based TLD-100 material (LiF:Mg, Ti), formed into chips. Other than achieving the sensitivity of TLD-100, previous studies [11] have demonstrated the ability of cylindrical cross-section Ge doped optical fibers to meet the main criteria required of a TL dosimeter (TLD), and thus attractive in TSL dosimetry other than at doses below those used in radiotherapy.

## 2. Materials and Method

### 2.1. Ge-Doped Flat Dosimeters and LiF Dosimeters

The fiber dosimeters studied herein were fabricated in the University of Malaya, at the Department of Electrical Engineering, using the fiber pulling tower located there. A review of the process followed in obtaining these flat fibers from the starting silica preform is covered in A. Shafiqah *et al*., Dambul *et al*., and Siti Abdul Sani *et al*., [9,12,13], it also being noted that in the process of the fiber drawing additional defects can be induced. The doped optical fiber samples, of nominal length 6 mm (obtained using an optical fiber cleaver), were of four specific cross-sectional dimensions: 85 × 270 µm, 100 × 350 µm, 165 × 620 µm and 200 × 750 µm, the larger dimension in each pairing corresponding to the length along the contact surface axis, while the smaller dimension corresponds to the thickness transverse to this contact surface. The mass of the fibers were shown in Table 1. Fifty samples of each cross-sectional value of optical fibers were obtained.

**Table 1:** Respective mass for each cross-sectional form of doped fiber.

Cross-Section Dimensions (µm)	Mass (mg)
85 × 270	0.21 ± 0.01
100 × 350	0.36 ± 0.01
165 × 620	1.19 ± 0.01
200 × 750	1.99 ± 0.01

The response of the fiber dosimeters have been compared against lithium fluoride TLD-100 chips (Thermo Fisher Scientific Inc, Waltham, MA, USA) of dimension 3.2 × 3.2 × 0.89 mm. In addition to being formed of LiF, this type of TLD chip also contains Li in the isotopic proportion ^6^Li (7.5%) and ^7^Li (92.5%). The mass of these TLD-100 samples was determined using an electronic balance, yielding a mean value of 23.6 mg.

For both forms of dosimeter, TLD-100 or fibers, the main purpose of carrying out calibration is to ensure that all dosimeters provide practically identical response to a given radiation dose. For this a so-called elemental correction coefficient (ECC) is obtained. Here one notes that variations in dopant concentration, most specifically along the length of the fibers, result from the fiber pulling process. The Element Correction Coefficient (ECC) for each dosimeter was obtained as:

(1) $$ECC_{j}\;=\;\frac{<TLD>}{TLD_{j}}$$

with <*TLD*> and *TLD_j_* representing the mean *TLD* value and individual reading values respectively.

### 2.2. Annealing

Prior to use of the thermoluminescent materials, annealing was carried out in order to erase any previous history of filled defects, including those arising from irradiation or tribo- and chemoluminescence, stabilizing the trap structure and restoring them to initial conditions. The samples were annealed for one hour in an oven maintained at a temperature of 400 °C. During annealing, the various media were positioned on a ceramic plate and then subsequent to annealing, to minimize thermal stress, the samples were slowly allowed to cool to ambient room temperature [1,2,3,4,5]. Upon completion of the preparation routine, for ease of storage, handling and irradiation, the samples were subsequently placed inside appropriately labelled gelatine capsules. When not in use, the fibers were kept in light-tight containment to avoid exposure to ambient light.

### 2.3. Samples Irradiation

For irradiation, use was made of a conventional ^60^Co Gammacell-220 self-shielded irradiator, the source activity at the time of irradiation providing a gamma-ray dose-rate of 0.0522 Gy/s. The first TL study concerned evaluation of the element correction coefficient (ECC) for each sample, essentially the individual sensitivity of each sample, irradiating the entire collection of fibers and TLD-100 chips, all to a dose of 10 Gy. For the subsequent sets of TL measurements, three samples of each cross-section of flat fiber together with TLD-100 chips were irradiated to doses ranging from 2.0 to 10.0 Gy, in increments of 2.0 Gy, allowing the linearity of dose response and reproducibility to be evaluated.

### 2.4. Samples Readout

The TLDs were readout using a Harshaw 3500 TLD reader, supported by WinREMS software, the TLD reader being supplied with a slow flow of nitrogen gas, in part to inhibit sample oxidation. The maximum temperature during data acquisition was 400 °C while the preheat temperature was 40 °C. The temperature ramp rate during signal acquisition was 10 °C·s^−1^. Optimum TLD reader settings were used. For each dosimeter, the TL readings were normalized to dosimeter mass, obtaining results in μC/mg.

## 3. Results and Discussion

### 3.1. TL Glow Curve

**Figure 2:**
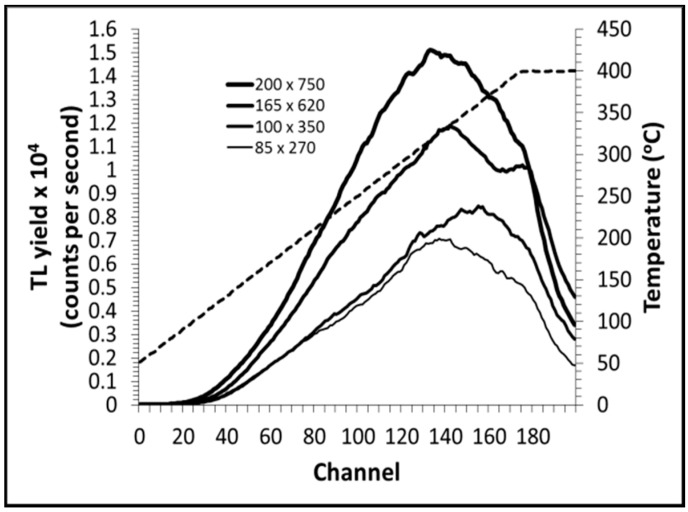
Typical fiber TL glow curves, all for a dose of 30 Gy delivered at Co-60 gamma-ray energies, encompassed between temperatures of ~200 °C and 370 °C. (Note: the dotted line represents the temperature-time profile for the heating rate used (as noted in the text), with the temperature scale on the right-hand vertical axis).

Figure 2 shows the glow curves for the various TL media studied herein, this being a display of intensity of luminescence as a function of temperature [1]. The detailed form of the glow curve varies with the particular heating cycle used and also the physical constitution of the sample, be it entirely crystalline, amorphous, or some mixture of these. For each flat fiber cross-section, a broad peaked structure is observed, the peak maxima occurring at temperatures within the range 320 °C to 370 °C, appearing approximately between the channel numbers 120–160. For the fibers, for each cross-section, the glow curves are typically similar in shape, although a double-peaked structure is observed for the flat fiber of cross-sectional dimensions 165 × 620 µm, potentially indicative of a microcrystalline component to the predominant amorphous medium. The area under each graph represents the number of electrons released from traps and, by association the radiation energy deposited, the glow peak maxima indicating the maximum in the number of electrons released from traps. In general, the most desirable glow curve for TLD is one of simple form (reflecting a simple trap distribution), allowing ease of interpretation. In the case of a more complex glow curve, the main peak (*i.e*., the dosimetric peak) should be well resolved from other possible peaks in the glow curve [5].

### 3.2. Dose Response

**Figure 3:**
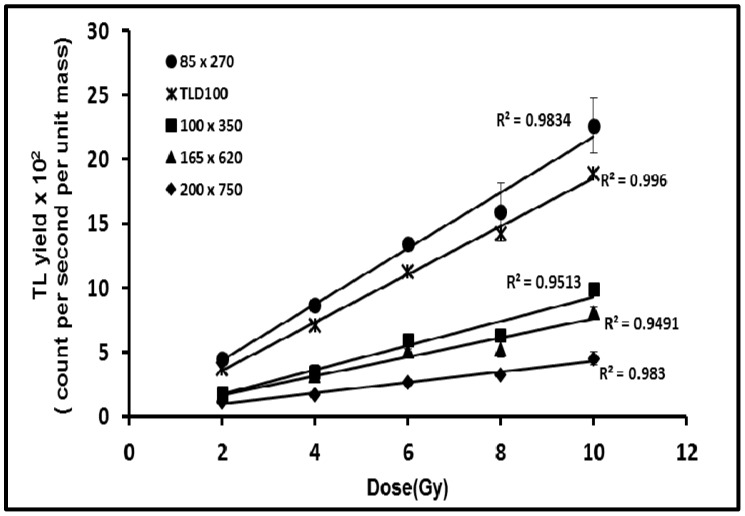
Dose response of Ge-doped optical fibers of cross-section dimensions 85 × 270 µm, 100 × 350 µm, 165 × 620 µm and 200 × 750 µm compared to that of TLD100, provided together with the standard error of the mean. The dotted lines are least square fits to the data, obtaining respective correlation coefficients of 0.983, 0.951, 0.949, 0.983, and 0.996. (Note: in some cases the error bars are smaller than the data points).

In the present study, the dose response of the fibers were analyzed over a range of doses for the various flat fibers, the latter being defined by their different cross-sectional dimensions. The fibers and TLD100 chips were irradiated to doses ranging from 2 to 10 Gy using a ^60^Co gamma-ray source, the doses being increased in increments of 2 Gy. Each experimental point represents the mean value obtained over five repeat measurements, normalized in each case to the mass of each fiber type. The error bars were obtained from the standard deviation of the readout for the five fibers used at each dose. The associated dose response curves (according with the integrated TL yield at each dose) for the various flat fiber cross-sections and for the TLD100 chips are shown in Figure 3, the linear TL response supporting findings from previous study [1,8,10,11,14]. In the present study, the increase in TL yield with dose remains linear over a wide range of values, from 2 Gy up to 10 Gy, the flat fibers of physical dimensions 200 × 750 µm, 165 × 620 µm, 100 × 350 µm, 85 × 270 µm showing TL yields of the order of 0.22, 0.40, 0.50 and 1.10 respectively with respect to TLD100 chips. The smallest size of fiber (85 × 270 µm) provides the greater TL yield, greater even than that of TLD100. Indeed, it is also apparent that there is a progressive improvement in TL yield, scaling as an inverse function of fiber cross-section dimensions (see below for further analysis of this). The result is supported by previous findings [8] using the same type of flat fiber and by other findings [10] comparing three forms of pure (undoped) fiber (capillary-, flat-, and photonic crystal fiber (PCF)) and two forms of Ge-doped fiber (capillary- and flat-fiber), the flattened form bringing about improvement in sensitivity. As an aside, it can further be noted that Benabdesselam *et al*. [15] have pointed to an additional sensitivity dependence of TSL response for Ge-doped silica fibers, significantly increasing with readout heating rate. Conversely, for the phosphor-based TLD-500 dosimeter, the TSL response decreases with increase in readout heating rate [15].

**Figure 4:**
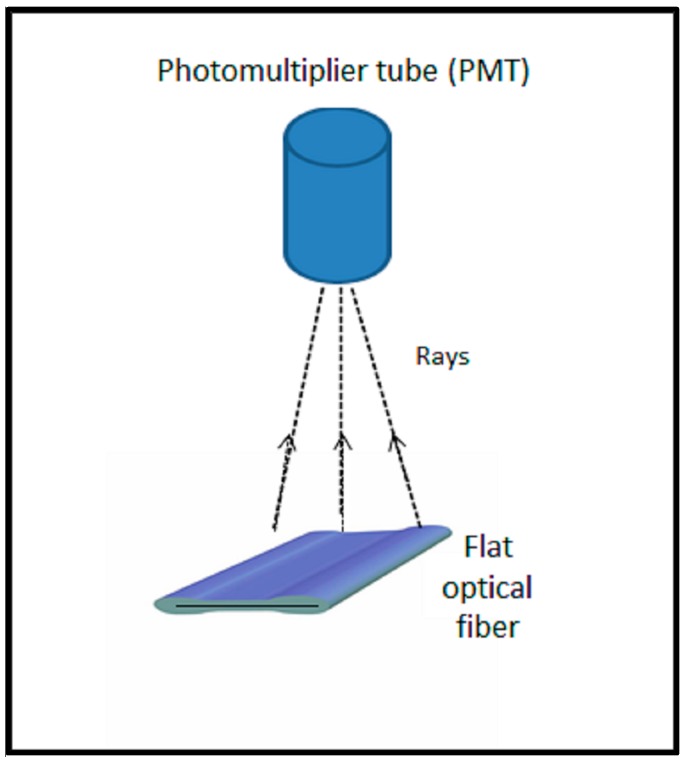
The position of flat optical fibers vie wed by a centrally located fixed position TLD Reader photomultiplier tube. (Note: the line on the flat optical fiber represents the core containing the Ge dopant).

**Figure 5:**
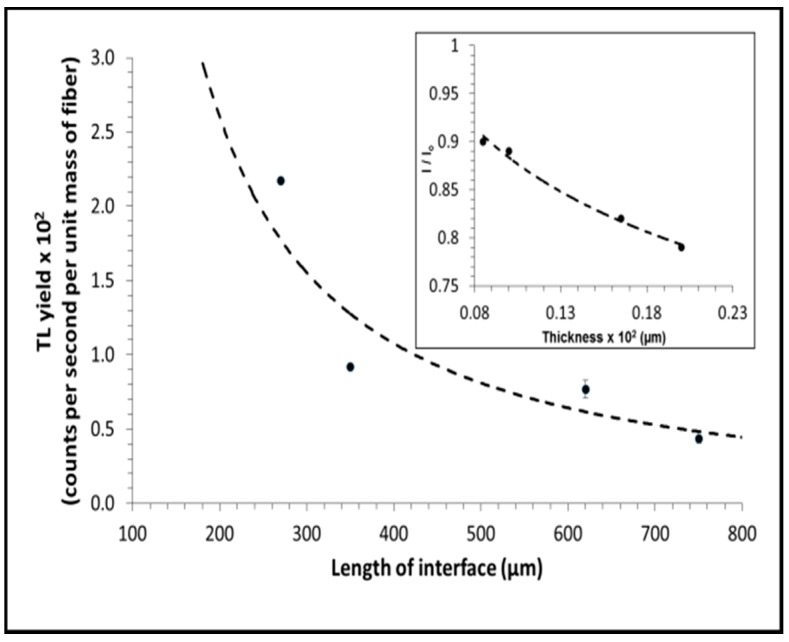
Calculated experimental TL yield per unit mass, mapped against the progressive increase in inner surfaces contact length. The inset shows an analysis of the effect of fiber transverse thickness on TL light transport, where I_0_ and I are the intensity (power per unit area) of the incident radiation and the transmitted radiation through the silica component of the fiber medium, based on a simple Beer-Lambert (monochromatic, single scattering) approximation.

In seeking to obtain an interpretation of the results of Figure 3, we investigate the observed TL yield per unit mass of the flat fibers, starting from the standpoint of a fixed concentration TL source (6 mol% Ge), with fabrication of the fibers being such that the doped core of the fibers become progressively thin as the cross-section dimensions of the fibers increase. In association with this is the observed increase in inner surfaces contact length of the flat fibers (hereafter referred to as the interfacial length). The arrangement is viewed using a centrally located fixed position TLD Reader photomultiplier tube, TL light along the central bisector to the longer of the two cross-section dimensions of the fiber being received normal to the upper surface of the fiber (Figure 4). The larger graph of Figure 5 provides a plot of the resulting TL yield, chosen here to be mapped against the interfacial length, showing an expected diminution in TL light yield, contributed to by (among other factors): increasing path length of light through the silica component of the fibers together with the associated multiple scattering (see below). For the largest cross-section fiber, the TL yield per unit mass reduces to ~25% of that of the smallest cross-section fiber. An intuitive analytic fit to the data is also provided, of the form *y* ∝ 1/*x* (TL intensity falling off inversely with interfacial length, with a constant of proportionality of ~0.2. The inset to Figure 5 provides an analysis of light photons attenuation for light of wavelength ~250 nm (this value according with defects of ~5 eV) transported through the irradiated silica as a function of transverse sample thickness in accord with a simple exponential attenuation (Beer-Lambert law) dependency. It is well known that germanium sites in silica have an absorption spectrum consisting of two strong singlet-singlet transitions near 242 nm and 180 nm. The absorption at 242 nm is strong for oxygen-deficient germanium-doped silica (e.g., with germanium-related oxygen-deficient centers (GODC’s) such as two-coordinated germanium) [16]. The value of linear attenuation coefficient is highly dependent on the wavelength of the light, but here one takes guidance from published data for highly irradiated silica, adopting a linear attenuation coefficient µ of 1.2 mm^−1^ for light of wavelength 250 nm [17]. It is apparent from the latter that light transmission through the fiber towards the photomultiplier tube of the TLD Reader would account for a loss of no more than 20% of initial TL yield. However, the practical situation is very much more complex than provided for herein, including a source of diminishing thickness embedded in silica of increasing thickness and lateral extent, with no account being made in the simple application of the Beer-Lambert law of the increasing probability for multiple scattering [a typically adopted value for single scattering events of 0.4 mfp (mean-free path) is usual, producing a value of 330 µm], inelastic scattering into longer wavelengths also being unaccounted for. It is apparent that, in seeking to account for the more substantial light loss, there is need for a more detailed model, also requiring analysis of self-absorption due to the Ge dopant.

### 3.3. Sensitivity

**Figure 6:**
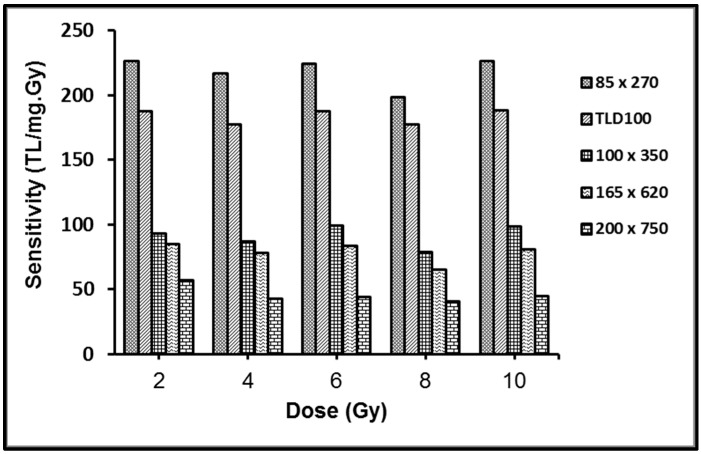
Sensitivities of the different dimension flat fibers and TLD100.

The sensitivities of the four different cross-sectional dimension flat fibers and TLD100 have been compared as shown in Figure 6. The relative sensitivities were determined to be 0.25, 0.42, 0.49, 0.3, and 1.19 for 200 × 750, 165 × 620, 100 × 350, and 85 × 270 µm respectively with respect to TLD100 chips. The results illustrate the 85 × 270 µm fiber to enjoy superior sensitivity to that of the larger dimension fibers as well as that of TLD100. 

### 3.4. Fading

**Figure 7:**
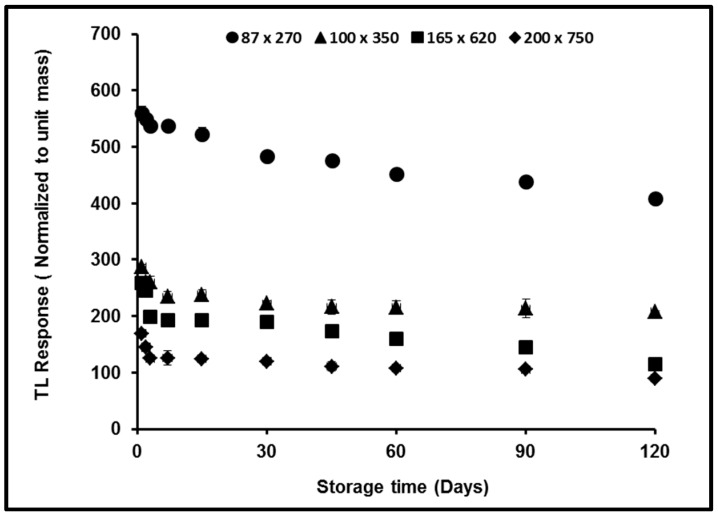
The loss of TL yield over a period of 120 days post-irradiation for the variously dimensioned Ge-doped flat fibers, irradiated to a dose of 2 Gy using the ^60^Co gamma source. (Note: In some cases the error bars are smaller than the size of individual data points).

Fading is one most important TL effects to be investigated in seeking to understand the loss of response post irradiation, also in evaluating the efficiency of any proposed TLD. In present research, the fading of TL signal displayed by the Ge-doped flat fibers was observed over a period of four months and shown in figure 7. In investigating the effect of absorbed dose on TL signal stability, 480 samples of the different dimension fibers and dopant concentrations were simultaneously irradiated using the ^60^Co gamma source. The samples were exposed to a dose of 2 Gy and then kept under darkened conditions at room temperature ~27 to 28 °C until readout. The smallest dimension fiber *i.e*., 85 × 270 µm displayed the lowest signal loss over the period of study, at around 26.9%. The mean loss in TL yield has been estimated to be 0.22%–0.46% per day. The fading effect of Ge-doped silica optical fibers obtained in this study is lower than the results obtained by TLD-700, which is 75.9% over six days [18,19].

### 3.5. Reproducibility

**Figure 8:**
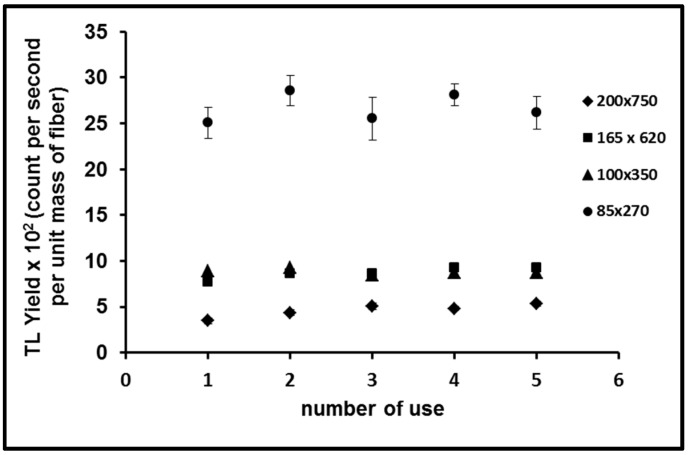
Reproducibility for the various Ge doped fibers exposed to doses of 10 Gy, provided together with the standard error of the mean.

In this study, three samples of each form of flat fiber were exposed to a dose of 10 Gy in a series of five repeat cycles of irradiation, measurement and annealing. The results are shown in Figure 8, the optical fibers showing good reproducibility with a standard deviation of less than 4%. Also apparent is that the data spread progressively increases with reduction in fiber cross-sectional dimensions. The as yet unpublished data of Abdul Sani (personal communication, 30 November 2014) for elemental concentration of dopant in fibers of various dimensions support this observation, with greater variability seen for the smaller core sizes.

## 4. Conclusions

Present research describes an investigation of gamma radiation response of Ge-doped SiO_2_ flat optical fibers of various cross-sections. The key TL parameters, glow curve, dose response, sensitivity, fading and reproducibility have been studied. The results clearly show the samples to be suitable for use as TL dosimeters, with good linearity of response, re-useable and with a simple glow curve *i.e*., a simple trap distribution. The greater TL yield per unit mass is obtained for the smaller dimension fibers, an observation supported by analysis of the effect of light transportation through fibers of various dimensions. 
